# Clinical Efficacy of Varying Dosages of Intravenous Phenylbutazone in a Reversible Model of Equine Foot Lameness

**DOI:** 10.3390/ani16193096

**Published:** 2026-10-02

**Authors:** Jonathan H. Foreman

**Affiliations:** Department of Veterinary Clinical Medicine, College of Veterinary Medicine, University of Illinois Urbana-Champaign, 1008 West Hazelwood Drive, Urbana, IL 61802, USA; jhf@illinois.edu

**Keywords:** horse, non-steroidal anti-inflammatory drug, NSAID, pain, analgesic

## Abstract

The objective of this experiment was to compare the clinical efficacy of various dosages of phenylbutazone (PBZ) with a negative control. The hypothesis was that higher PBZ doses would result in improved efficacy in a dose-dependent manner. Eight horses were shod with adjustable heart bar shoes on one front foot. Grade 4.0/5.0 lameness was induced every 2 to 4 weeks by tightening a set screw against the heart bar. Heart rate (HR) and lameness score (LS) were monitored at rest; every 20 min after lameness induction for 5 h; and hourly for another 8 h. One hour after lameness induction, treatment was administered IV in a randomized blinded manner and included negative control (isotonic saline: SAL) or PBZ at 2.2 (LOW), 4.4 (MOD), or 8.8 (HIGH) mg/kg body weight. Jugular blood samples were obtained at experiment hours 0, 1:05, 3, 5, 9, and 13, and were evaluated for plasma PBZ concentration. Results were compared using repeated measures analysis of variance and Student–Newman–Keul’s test with the level of significance set at *p* < 0.05. MOD and HIGH dose PBZ reduced HR from 3.0 h through 12.0 h and LS from 1.67 h through 12.0 h post-administration (*p* < 0.05); there was no difference in response between MOD and HIGH dose PBZ. Compared to SAL, LOW dose PBZ reduced HR from 3.67 h through 5.0 h and LS from 3.67 h through 4.0 h after administration (*p* < 0.05). The conclusions were that LOW dose PBZ was clinically effective for only a brief period compared to SAL, and HIGH dose was not more effective than MOD dose PBZ.

## Simple Summary

Despite its widespread use as an anti-inflammatory medication in horse practice, varying dosages of phenylbutazone (PBZ) remain poorly studied. It is taken for granted commonly that lower dosages have less pain-killing effect, and that higher dosages have greater effect, but there are no well-designed published studies documenting the dosage effect. On a rotating basis every 2 to 4 weeks, we used an adjustable heart bar shoe to induce reversible lameness in 8 horses. After 1 h of lameness, we administered a moderate dosage of PBZ (MOD: 4.4 mg/kg), half that dosage (LOW), double that dosage (HIGH), or no dose (saline placebo: SAL) intravenously and then monitored the horses for 12 h after treatment. Heart rate and lameness score were the primary monitoring variables assessed by an investigator blinded to treatment assignment. For both variables, LOW was considerably less effective than MODERATE, but HIGH was no more effective than MOD, when treatments were compared to SAL. The important findings were that LOW doses were less effective and were not as beneficial in relieving pain, but HIGH doses were not more effective than MOD doses and may be more toxic to the stomach, colon, and kidney of the horse.

## 1. Introduction

Phenylbutazone (PBZ) has been used widely as an anti-inflammatory and analgesic in equine medicine since the 1950s [1]. It acts through non-selective cyclooxygenase inhibition, resulting in a decrease in prostaglandin synthesis, thereby decreasing inflammation and fever if present. The conventional higher end of most label dosages is 4.4 mg/kg IV or PO once or twice daily. It is metabolized in the liver into various compounds, of which oxyphenbutazone and γ-hydroxyphenylbutazone retain the anti-inflammatory properties of the parent compound. PBZ is renally-excreted primarily as its metabolites with a minor portion excreted in bile [1]. Toxicity concerns include gastrointestinal ulceration and/or renal disease, especially when administered in the face of dehydration or other potentially nephrotoxic drugs like aminoglycoside antibiotics [2,3,4,5,6,7]. The label specifically states not to use PBZ for more than 5 consecutive days IV, due to the risk of renal disease and gastrointestinal ulceration. Practicing veterinarians mitigate toxicity concerns by shortening duration of usage, decreasing dosage, switching to oral administration, and ensuring proper hydration, if indicated, with IV or oral fluids.

Veterinarians often administer dosages lower than 4.4 mg/kg IV for a variety of reasons. They may feel that a lower dose is adequate for that individual horse’s need for analgesia; that is, that its condition is milder and may not require the full dose of 4.4 mg/kg. They may be attempting to mitigate the potential side effects of PBZ use, such as gastric or colonic ulceration or renal disease. They may be using a lower dose to mitigate the negative systemic effects of potential or fulminant endotoxemia such as with diarrhea, colic, pneumonia, pleuritis, or other Gram-negative bacterial infections [8,9,10]. Or they may be attempting to avoid a positive drug test result by using a lower dose. However, there are minimal published data on the analgesic efficacy of doses lower than 4.4 mg/kg IV.

The relationship between injury and elevated HR is well-documented in human runners [11] and swimmers [12]. Previous research in our laboratory has shown heart rate (HR) to be elevated with lameness before, during, and/or after exercise when compared to negative controls in laboratory models [13,14,15,16,17,18,19,20,21] and in field Thoroughbred training settings [22]. These HR elevations increase with increasing degrees of lameness in our model where lameness is induced by tightening a set screw into an adjustable or mechanical heart bar shoe [23,24,25], thereby causing the heart bar to apply temporary painful pressure to the frog of the foot [13,14,15,16,17,18,19,20,21]. The pain created in this model is quickly reversible with loosening of the shoe and has been shown repeatedly to be NSAID-responsive [13,14,15,16,17,18,19,20,21].

Despite the decades-long and widespread use of PBZ in equine medicine, there is a dearth of objective data on the effects of varying IV dosages. The objective of this experiment was to compare the clinical efficacy of various IV dosages of PBZ with a negative control placebo. The hypothesis was that use of higher PBZ IV doses would result in improved efficacy in a reversible model of foot lameness compared to a negative control.

## 2. Materials and Methods

All animal procedures were conducted in accordance with applicable institutional and national guidelines for the care and use of animals and were reviewed and approved by the University of Illinois Urbana-Champaign Institutional Animal Care and Use Committee.

**Subjects**—Eight horses (5 Thoroughbreds, 2 Quarter Horses, 1 Morgan; 4 mares, 4 geldings) were studied for 4 weeks. Mean (±SE) age was 9.3 ± 2.4 years (median 5 years, range 3–20 years old). Mean body weight was 442.7 ± 22.1 kg (median 466.6 kg, range 380.5–571.4 kg). Complete physical and lameness examinations were performed before shoeing to ensure that each subject was normal before experiments were begun. Each subject’s left front foot had an adjustable heart bar shoe applied by one experienced farrier. The right front foot had a simple keg shoe of a similar weight applied for balance. A minimum of 7 days of stall rest were allowed after shoeing and before any trials were begun.

**Lameness induction**—Lameness was induced by tightening a set screw against the adjustable heart bar 1 h prior to administration of treatment. Cadaver Instron-testing data have documented that tightening of the mechanical heart bar shoe concavely deforms the frog, sole, and distal phalanx, likely provoking an inflammatory response in the soft and bony tissues of the foot [18]. Lameness was graded by one blinded investigator (J.H.F.) using a previously-described decimal grading system [13,14,15,16,17,18,19,20,21] based initially on the American Association of Equine Practitioners (AAEP) Lameness Grading Scale [26]. The addition of the intermediate steps in the decimal system allows use of fractions of grades in a continuous variable grading scheme. The use of an in-stall grading system is necessary in our model because of the reliance on HR as another continuous variable; if horses were to be jogged intermittently, that exercise would impact negatively on the ability to use HR as a physiological variable reflective of pain in the horse. Grades modified from the AAEP scale were 0.0 (sound or undetectable lameness), 1.0 (barely detectable lameness; horse intermittently looked lame at a walk in the stall and/or pointed the lame toe forward intermittently and rarely), 2.0 (mild lameness; horse was more consistently lame at a walk in the stall, had a mild head bob when walking in the stall, and pointed its toe more consistently), 3.0 (moderate lameness but not non-weight-bearing; horse had more obvious head bob at a walk, toe pointing more frequently), 4.0 (non-weight-bearing 50% of the time, severe head bob, toe pointing whenever not walking but not always three-legged lame at a walk), and 5.0 (non-weight-bearing 100% of the time) [13,14,15,16,17,18,19,20,21]. A lameness grade of 4.0 was achieved initially in each subject when undergoing a drug trial.

**Treatments**—Treatments were administered 1 h after lameness induction and included: moderate-dose PBZ (MOD: 4.4 mg/kg IV) (Butler Company, Columbus, OH, USA), lower-dose PBZ (LOW: 2.2 mg/kg IV), higher-dose PBZ (HIGH: 8.8 mg/kg IV), and isotonic saline (SAL: 1 mL/45 kg IV, yielding a volume similar to that when MOD dose PBZ is administered, 10 mL to a typical 1000 lb or 450 kg adult horse) (0.9% Sodium Chloride Solution U.S.P., 1000 mL, Hospira, Inc., Lake Forest, IL, USA). Treatments were assigned randomly to the 8 subjects in a blinded balanced Latin Square Design replicated twice. Two horses received each of the 4 treatments each test day. On subsequent treatment days, horses remained in their assigned pairs but received a different treatment than previously based on a rotation of treatment assignments. Treatments were administered by one co-investigator (K.K.S.) who was responsible for protecting the secrecy of treatment assignments to ensure that the principal investigator (J.H.F.) measuring HR and lameness score (LS) remained blinded throughout the experiment. The assignment code was not broken until after all treatment days were completed. Treatment days were a minimum of 17 days apart to ensure washout of the previously-administered treatment.

**Monitoring**—HR and LS were assessed in the stall every 20 min for the first 5 h of each lameness trial and then hourly for an additional 8 h (for a total of 13 monitoring hours each treatment day). Baseline HR and LS were established by monitoring during the first hour after lameness induction but before PBZ or SAL administration. Drug or placebo was administered 1 h after lameness induction, and horses were monitored for 12 h after drug administration. These monitoring techniques have been used successfully previously in our laboratory [13,14,15,16,17,18,19,20,21].

**Phenylbutazone analysis**—Jugular venous blood samples were obtained via 20-gauge needle into a heparinized vacuum tubes (Vacutainer, Becton Dickinson, Franklin Lakes, NJ, USA) at experiment hours 0 (pre-treatment), 1:05 (5 min after treatment, representing peak plasma concentration), 3, 5, 9, and 13 (12 h after treatment, representing trough plasma concentration) and were evaluated for plasma PBZ concentration. Samples were frozen until processing after completion of all treatment assignments. Plasma PBZ concentrations were determined in an external laboratory by use of high-performance liquid chromatography (HPLC) with a lower limit of detection (LLD) for PBZ of <1.0 µg/mL [15,27]. Drug plasma samples (5 mL) were adjusted to pH 5.0 with dilute HCl, and PBZ was extracted in 2 × 8 mL chloroform: isopropanol (99:1). After evaporating to dryness, samples were reconstituted with 200 µg of methanol. Aliquots were then measured by HPLC for plasma PBZ concentrations [15,27]. Known control samples were used to validate the method using serially-diluted plasma samples obtained from untreated horses. The drug-free plasma samples were spiked with PBZ to create known plasma concentrations from 1.0 through 50.0 µg/mL of PBZ. Samples were masked by collection time and dose administered but not to drug sought (PBZ). Intra- and inter-assay precision and accuracy coefficients of variation were <5% at the LLD on a single column calibrated in triplicate.

**Statistical analysis**—Based on previous studies, 8 horses were sufficient for this design [13,14,15,16,17,18,19,20,21]. Power calculation documented that a sample size of 8 horses was adequate for this experiment with an expected HR of 50/min for SAL and 36/min for MOD treatments with mean HR SD of ≤10% and alpha 1 set at 0.05 and 80% power (https://clincalc.com/, Rosalind Franklin University, North Chicago, IL, USA).

Mean (±SE) HR, LS, and plasma PBZ concentration were determined for each treatment at each sampling interval. LS was treated as a continuous variable since a multi-stepped decimal grading scale was used [28,29,30]. Many statisticians now agree that ordinal scales with multiple steps, particularly those with 10 or more steps, represent continuous data and should be treated as such for purposes of statistical analysis [28,29,30]. Furthermore, it has been stated that “parametric tests are generally more robust that nonparametric tests” [29]. Significance of differences between treatments was tested by repeated measures analysis of variance (RM ANOVA) (SigmaPlot 11.0, Systat Software, Inc., San Jose, CA, USA). Prior to conducting RM ANOVA, normality was assessed using the Shapiro–Wilk test. When normally distributed, if RM ANOVA then indicated significant time-by-treatment interactions, differences at specific sampling intervals were tested by Student–Newman–Keul’s test. A value of *p* < 0.05 was considered significant.

## 3. Results

Statistical diagnostics confirmed that the parametric assumptions for the RM ANOVA were satisfied. The model residuals were normally distributed (Shapiro–Wilk test, *p* > 0.05). When the LS data were analyzed using the Wilcoxon signed-rank test, a nonparametric test designed for ordinal, not continuous data, the same results were obtained.

**Figure 1:**
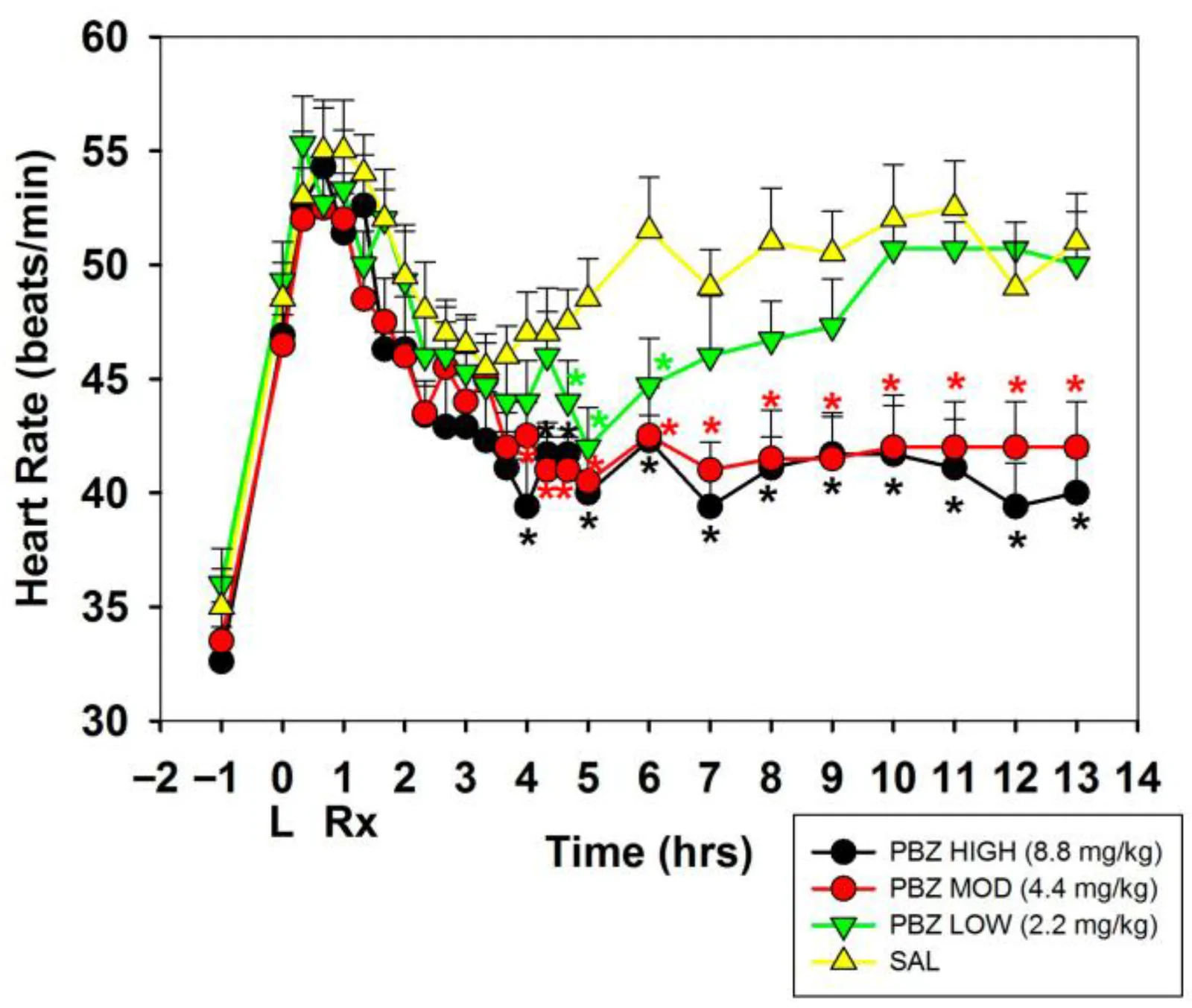
Mean (±SE) heart rate (beats/min) versus time (hours) for 12 h after treatment was administered at Hour 1 (designated by Rx). Lameness was induced at Hour 0 (designated by L). When compared to SAL negative control, lameness-induced heart rate elevation decreased for both MOD dose (4.4 mg/kg) and HIGH dose (8.8 mg/kg) PBZ from 3.0 h through 12.0 h post-administration (red and black asterisks, respectively: *p* < 0.05). Heart rate response to treatment was not different between MOD and HIGH treatments (*p* > 0.05). LOW dose PBZ (2.2 mg/kg) reduced LS compared to SAL only from 3.67 h through 5.0 h after administration (green asterisks: *p* < 0.05).

**Figure 2:**
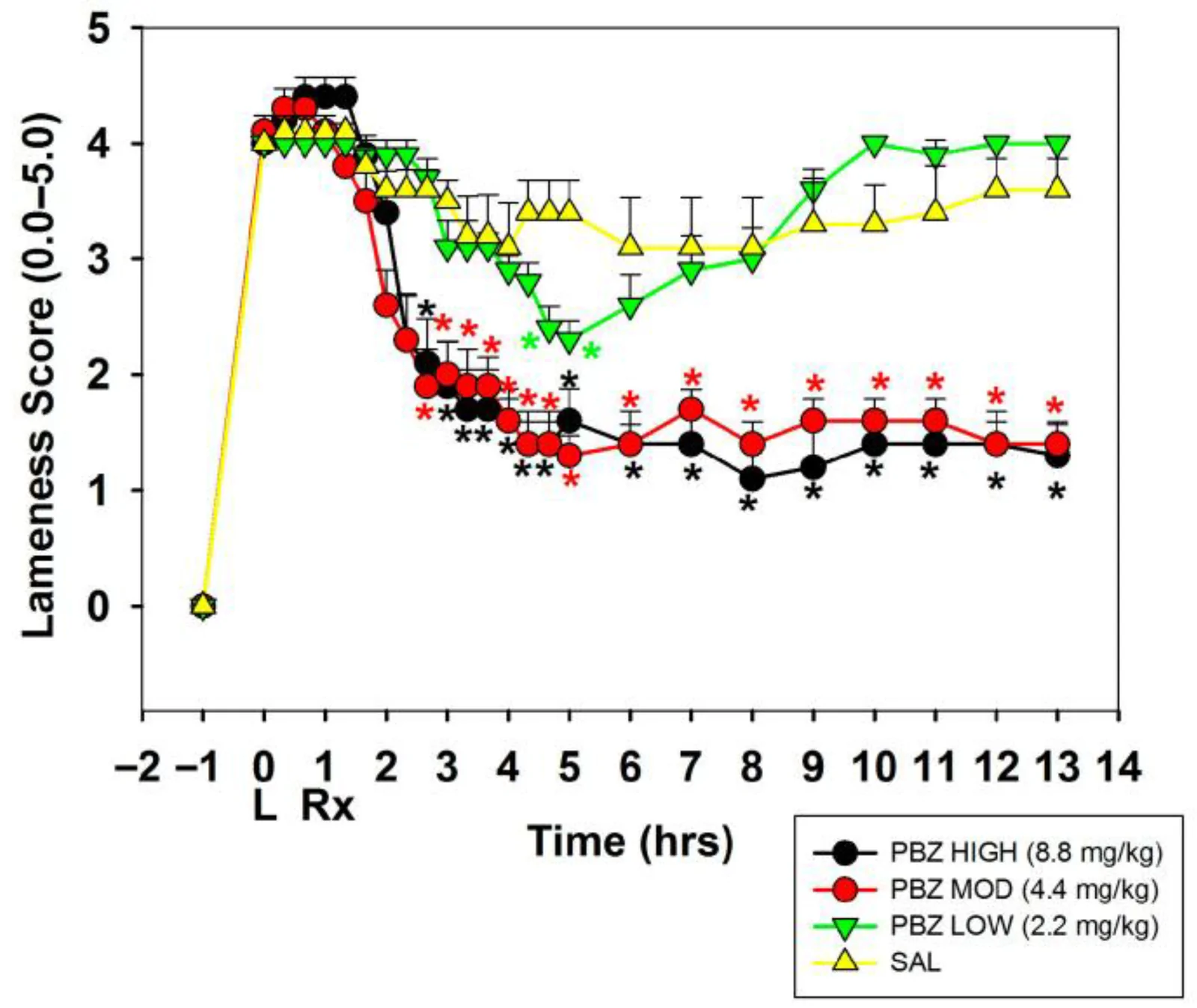
Mean (±SE) lameness score (LS) versus time (hours) for 12 h after treatment was administered at Hour 1 (designated by Rx). Lameness was induced at Hour 0 (designated by L). When compared to SAL negative control, LS decreased for both MOD (4.4 mg/kg) and HIGH dose (8.8 mg/kg) PBZ from 1.7 h through 12.0 h post-administration (red and black asterisks, respectively: *p* < 0.05). LS response to treatment was not different between MOD and HIGH dose treatments (*p* > 0.05). LOW dose PBZ reduced LS compared to SAL only from 3.67 h through 4.0 h after administration (green asterisks: *p* < 0.05). LS was not different between LOW dose PBZ and SAL from 5.0 h through 12.0 h after treatment (*p* > 0.05).

**Figure 3:**
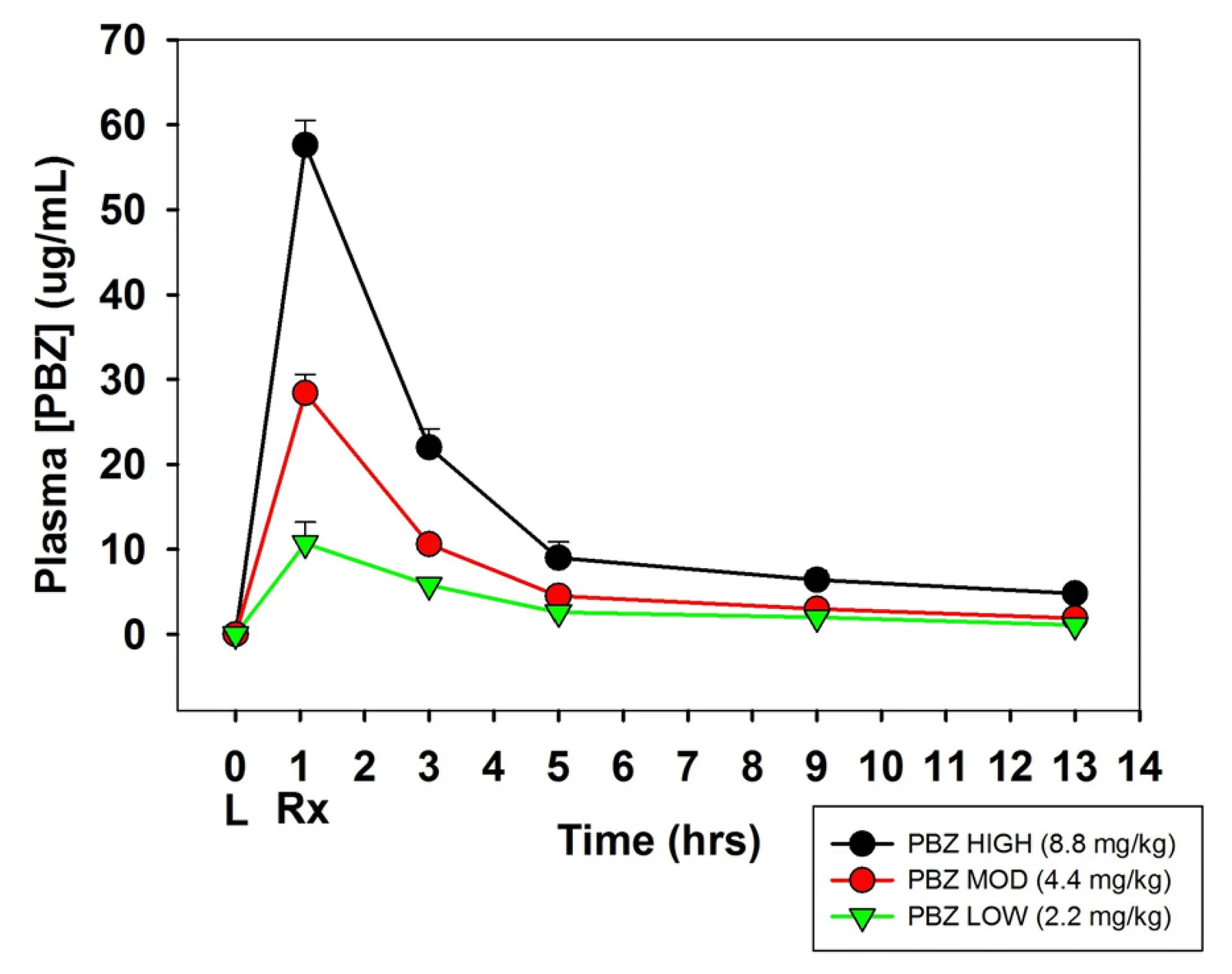
Mean (±SE) plasma PBZ concentration (µg/mL) versus time (hours) for 12 h after treatment was administered at Hour 1 (designated by Rx). Lameness was induced at Hour 0 (designated by L). Peak samples were obtained from jugular blood samples taken at 1:05 h after lameness was induced or 5 min after IV injection. Trough samples were obtained 12 h after IV PBZ injection (Hour 13 on the *x*-axis).

HR and LS changes mirrored one another. HR, LS, and PBZ plasma concentrations are depicted graphically in Figure 1, Figure 2, and Figure 3, respectively. In each of the graphs, lameness was induced at 0 h (designated by L) on the *x*-axis (time) in the graphs. All treatments were administered at 1 h on the *x*-axis (time) in Figure 1, Figure 2 and Figure 3 (designated by Rx).

### 3.1. Heart Rate (Figure 1)

MOD and HIGH PBZ reduced HR from 3.0 h through 12.0 h post-administration (*p* < 0.05) (Figure 1). There was no difference in HR response between MOD and HIGH PBZ (*p* > 0.05). LOW PBZ reduced HR compared to SAL only from 3.67 h through 5.0 h after administration (*p* < 0.05). HR was not different between LOW PBZ and SAL from 6.0 through 12.0 h after treatment (*p* > 0.05).

### 3.2. Lameness Score (Figure 2)

MOD dose and HIGH dose PBZ reduced LS from 1.7 h through 12.0 h post-administration (*p* < 0.05). There was no difference in LS response between MOD and HIGH dose PBZ (*p* > 0.05). Compared to SAL, LOW dose PBZ reduced LS only from 3.67 h through 4.0 h after administration (*p* < 0.05). LS was not different between LOW dose PBZ and SAL from 5.0 h through 12.0 h after treatment (*p* > 0.05).

### 3.3. Plasma PBZ Concentration (Figure 3)

Mean plasma peak concentration 5 min after IV injection were 57.6 ± 2.0, 28.4 ± 2.2, and 10.7 ± 2.5 µg/mL plasma for HIGH, MOD, and LOW administrations, respectively. Concentration of all three dosages decayed exponentially until the trough samples 12 h after administration averaged 4.8 ± 0.6, 1.9 ± 0.2, and 1.1 ± 0.1 µg/mL, respectively.

## 4. Discussion

Despite its use in horses for over 65 years, there is a dearth of objective controlled data regarding the clinical efficacy of varying IV doses of PBZ in lame horses. For both principal variables (HR and LS), HIGH was not more efficacious than MOD PBZ, but LOW was less efficacious than MOD PBZ. These data show clearly that the analgesic effect of PBZ has a dose effect, with MOD doses more effective than LOW doses. It is important for veterinary practitioners to realize from these data that LOW doses of PBZ have limited measurable clinical efficacy in equine foot lameness, being essentially non-efficacious for the second half of the 12 h interval after dosing in this experiment.

Conversely, the use of HIGH doses of PBZ in this experiment was no more efficacious than were MOD doses. This finding is also important for veterinary practitioners who often are challenged by patients or clients to provide more analgesia for horses with musculoskeletal soreness. While it may be tempting to use larger doses to provide more analgesia, clearly the higher dose studied here was no more efficacious than the typical single dose. However, the toxicity of NSAIDs is drug-, dose- and duration-dependent [2,3,4,5,6,7], so use of a higher dose should at least be more efficacious to justify the added risk for toxicity. These data demonstrate that there is no analgesic benefit to use of the higher dose. Some users have argued that the higher dose allows efficacy to last longer than the typical 12 h window (J.H.F., personal communication, 1985–2026), but the risk of greater toxicity mitigates against this practice. Furthermore, the plasma PBZ concentrations (Figure 3) document that by 12 h after administration, there were detectable but limited differences in the plasma concentrations despite the different dosages. Practitioners must be cautious and judicious in the NSAID doses they choose to administer, and based on these data, there is no value to the use of HIGH doses of PBZ in foot pain modeled using the shoeing system studied here.

It is important to acknowledge that Keegan et al. (2008) previously reported some efficacy with 5 days of LOW doses of oral PBZ alone [31], but they did not perform, nor was their study designed to perform, a dose titration study as we have presented here. In their study of combined PBZ and FM use, they used 5 days of twice daily LOW dose oral PBZ (2.2 mg/kg) accompanied by twice daily MOD doses of FM IV (1.1 mg/kg) and showed increased efficacy of the combination over the LOW dose of oral PBZ alone [27]. We also demonstrated here some efficacy with IV, rather than their oral, LOW dose PBZ, but LOW efficacy was demonstrable for only a brief period of time after a single IV injection when compared to the saline negative control.

In a previous study of the efficacy of varying dosages of FM, we showed that HIGH dose FM (2.2 mg/kg IV) was no more efficacious than MOD dose (1.1 mg/kg IV) FM [16]. LOW dose FM (0.55 mg/kg IV) was less effective than MOD dose, but surprisingly, LOW dose was better than the negative control SAL for most of the 12 h post-treatment interval [18]. Interestingly, in an experimental model of endotoxemia, the effects of FM in minimizing the signs of endotoxemia were also dose-dependent, and FM was efficacious even at a quarter-dose preventatively (0.25 mg/kg IV) [9]. Despite PBZ being cited typically as more efficacious than FM for musculoskeletal pain in horses ([32], J.H.F., personal communication, 1990–2026), the LOW dose of FM persisted longer in reducing HR and LS in the previous study [18] than did the LOW dose of PBZ in the current study. Admittedly these are two different studies performed at different times with different horses, so comparison between the two studies must be done cautiously, but FM performed well in this model, even at LOW doses. However, with either PBZ or FM, the reduced dosage had measurable but limited efficacy in this model of foot lameness, especially compared to the MOD dose or HIGH dose treatments, and practitioners should be cautious about using reduced dosages in horses with severe foot pain.

Governing jurisdictions for equestrian competitions have widely-varying regulations regarding the use of NSAIDs before or on the day of competition. For over 40 years, the Fédération Equestre Internationale (FEI) has had a zero-tolerance policy for the detectable presence of any NSAIDs on competition day. Both PBZ and FM are prohibited within 6 days of FEI competition. Under the more liberal United States Equestrian Federation (USEF) rules, horses are prohibited from receiving any NSAID closer than 12 h prior to competition, with an allowable plasma PBZ concentration of <15 µg/mL. In the past, racing states in the USA varied in their allowance concentrations for NSAIDs in plasma on race day [33], with minimal but allowable plasma limits ranging from <5 µg/mL in many states to the stricter <2 µg/mL limit in Illinois. More recently, the new USA federal law entitled the Horserace Integrity and Safety Act (HISA) has classified PBZ as a “controlled substance,” allowing minimal concentrations <0.2 µg/mL on race day [34]. Our data in this experiment show that, with trough mean PBZ ranging between 1.1 ± 0.1 (LOW) and 4.8 ± 0.6 (HIGH) µg/mL, horses in this study at these dosages would still test positive under FEI or HISA rules but would be legal to compete under USEF rules. This continuing discipline- and association-specific variability in NSAID rules invites criticism of equestrian sports and challenges to their social license to operate.

Sadly, as trainers and veterinarians attempt to avoid positive plasma drug tests for NSAIDs during competition by minimizing the doses which they use prior to competition, they may sometimes be using non-therapeutic or minimally-therapeutic dosages, inadvertently providing little-to-no analgesia but still risking gastrointestinal or renal toxicity. The bottom line must be that if the horse’s condition requires analgesia, then it also requires an appropriate diagnosis followed by a therapeutic dose and an appropriate withdrawal time before it can compete under racing and most horse show association rules. The data in this study show that LOW doses of PBZ were efficacious but for less than half the time of MOD doses.

Limitations of this study included the number of subjects; more horses, where subjects are often limited in number, are nearly always better than fewer horses. Conversely, IACUC guidelines for an experiment where pain is induced encourage the use of a minimum number of subjects. Furthermore, we studied the efficacy of only one IV dose before ending each lameness trial, meaning we could have missed efficacy beyond the 12 h post-treatment window. Since this study we have completed 24 h long trials using drugs which purport to have 24 h efficacy, but we have never been comfortable using the induced lameness model for days at a time. Those longer-term studies might be carried out more acceptably using cases of spontaneous clinical disease [32], but they are then subject to greater inter-individual variability in initial presentation and progression of disease. Inevitably the use of a lameness grading system is sometimes characterized as subjective in the face of more recently adopted objective lameness scoring systems. However, the in-stall design is critical to the objective use of HR as a continuous variable in this model. The degree of lameness induced in this study was high, making the detection of lameness a simple process not in need of an IMU-based sensor system. Recent data have characterized visual observation of lameness as having a good correlation with a gait analysis system [35]. The LS system used here has been used in multiple experiments with success [13,14,15,16,17,18,19,20,21] but it is limited by the use of a single blinded observer and the absence of an independent objective lameness measure. Our lameness scale is perhaps more analogous to the pain ethograms proposed more recently for use for ridden horses manifesting various behavioral, facial, and bodily tics or stereotypies which can be used cumulatively to visually assess horses potentially manifesting pain [36,37].

## 5. Conclusions

From these data it was concluded that: (1) HIGH dose PBZ was no better in efficacy than was MOD dose PBZ (*p* > 0.05); (2) both MOD and HIGH dose PBZ improved HR and LS from 3.0 h or 1.67 h, respectively, through 12.0 h post-administration (*p* < 0.05); (3) LOW dose PBZ was efficacious for only a brief period of time (3.67–5.0 h for HR, 3.67–4.0 h for LS, post-administration) compared to the negative SAL control (*p* < 0.05).

## Data Availability

The original contributions presented in this study are included in the article. Further inquiries can be directed to the corresponding author.

## Abbreviations

The following abbreviations are used in this manuscript:

PBZ	Phenylbutazone
FM	Flunixin meglumine
HR	Heart rate
LS	Lameness score
SAL	Saline
NSAID	Non-steroidal anti-inflammatory drug
IV	Intravenous
SE	Standard error
HPLC	High-performance liquid chromatography
FEI	Fédération Equestre Internationale
USEF	United States Equestrian Federation
HISA	Horserace Integrity and Safety Act
PO	Per os, or orally
SD	Standard deviation
